# Neuropilin-1 antagonism in human carcinoma cells inhibits migration and enhances chemosensitivity

**DOI:** 10.1038/sj.bjc.6605539

**Published:** 2010-01-19

**Authors:** H Jia, L Cheng, M Tickner, A Bagherzadeh, D Selwood, I Zachary

**Affiliations:** 1 Centre for Cardiovascular Biology and Medicine, Department of Medicine, The Rayne Building, University College London, 5 University Street, London WC1E 6JJ, UK; 2 Ark Therapeutics Limited, The Rayne Building, University College London, 5 University Street, London WC1E 6JJ, UK; 3 Biological and Medicinal Chemistry Group, Wolfson Institute for Biomedical Research, University College London, Gower Street, London WC1E 6BT, UK

**Keywords:** EG3287, VEGF, integrin, migration, chemosensitivity

## Abstract

**Background:**

Neuropilin-1 (NRP1) is a non-tyrosine kinase receptor for vascular endothelial growth factor (VEGF) recently implicated in tumour functions.

**Methods:**

In this study we used a specific antagonist of VEGF binding to the NRP1 b1 domain, EG3287, to investigate the functional roles of NRP1 in human carcinoma cell lines, non-small-cell lung A549, kidney ACHN, and prostate DU145 cells expressing NRP1, and the underlying mechanisms involved.

**Results:**

EG3287 potently displaced the specific binding of VEGF to NRP1 in carcinoma cell lines and significantly inhibited the migration of A549 and ACHN cells. Neuropilin-1 downregulation by siRNA also decreased cell migration. EG3287 reduced the adhesion of A549 and ACHN cells to extracellular matrix (ECM), and enhanced the anti-adhesive effects of a *β*1-integrin function-blocking antibody. EG3287 increased the cytotoxic effects of the chemotherapeutic agents 5-FU, paclitaxel, or cisplatin on A549 and DU145 cells, through inhibition of integrin-dependent cell interaction with the ECM.

**Conclusions:**

These findings indicate that NRP1 is important for tumour cell migration and adhesion, and that NRP1 antagonism enhances chemosensitivity, at least in part, by interfering with integrin-dependent survival pathways. A major implication of this study is that therapeutic strategies targeting NRP1 in tumour cells may be particularly useful in combination with other drugs for combating tumour survival, growth, and metastatic spread independently of an antiangiogenic effect of blocking NRP1.

Neuropilin-1 (NRP1) is a co-receptor for class-3 semaphorins in neuronal guidance, and for the angiogenic cytokine vascular endothelial growth factor (VEGF or VEGF-A) in vascular development (Geretti *et al*, 2008; Pellet-Many *et al*, 2008; Larrivée *et al*, 2009). Neuropilin-1 requires plexin-A1 to transduce semaphorin-3A signalling in neuronal cells, which is implicated in chemorepulsion and neuronal cell migration (He and Tessier-Lavigne, 1997; Kolodkin *et al*, 1997; Takahashi *et al*, 1999; Tamagnone *et al*, 1999). In endothelial cells, NRP1 enhances VEGFR-2-mediated VEGF functions, including cell migration and angiogenesis (Soker *et al*, 1998; Whitaker *et al*, 2001; Lee *et al*, 2002). Neuropilin-1 and the closely related protein, NRP2, share 44% amino-acid sequence identity and a common structure comprising a large extracellular region containing a1/a2 (CUB), b1/b2 (FV/FVIII), and c (MAM) domains; a transmembrane domain; and a short cytoplasmic region (Chen *et al*, 1997; Kolodkin *et al*, 1997; Fujisawa, 2004). The a1/a2 and b1/b2 domains of NRP1 form the binding sites of semaphorin-3A, whereas VEGF binding requires the b1/b2 domain (Gu *et al*, 2002; Mamluk *et al*, 2002). The cytoplasmic domain of NRP1 consists of 44 amino acids and contains a C-terminal three-amino-acid PDZ-domain-binding motif, SEA, which binds to the PDZ domain protein, GAIP-interacting protein, at the C-terminus (GIPC), also called the neuropinlin-interacting protein (Cai and Reed, 1999). A naturally truncated and secreted form of soluble NRP1 splice variant has been identified (Gagnon *et al*, 2000), which maintains the binding properties (a and b domains) of full-length transmembrane NRP1.

Neuropilin-1 and NRP2 are present in various tumour types from patient specimens and overexpressed NRP1 or both NRPs correlate with tumour growth, disease progression, and patient prognosis (Bielenberg *et al*, 2006; Ellis, 2006; Guttmann-Raviv *et al*, 2006). Clinical studies of patients have shown that NRPl overexpression is positively associated with metastatic potential, advanced stage, and clinical grade of prostate carcinoma (Latil *et al*, 2000). In gastrointestinal carcinomas, increased expression of NRP1 correlates with the acquisition of invasive behaviour and metastatic potential (Hansel *et al*, 2004). Advanced colorectal carcinoma patients with high levels of NRP1 expression have shown a higher incidence of lymph node or liver metastasis and a shorter 5-year survival rate (Ochiumi *et al*, 2006). Coexpression of NRP1 and NRP2 also increases in the progression from dysplasia to microinvasive lung carcinoma, and correlates significantly with tumour progression and poor prognosis in patients with non-small-cell lung carcinoma (Kawakami *et al*, 2002).

Neuropilin-1 and NRP2 are expressed in a wide variety of human tumour cell lines and implicated in the survival, migration, and invasion of tumour cells (Bielenberg *et al*, 2006; Guttmann-Raviv *et al*, 2006; Frankel *et al*, 2008). It has been suggested that NRP1 predominantly expresses in carcinoma cell lines (epithelial origin), including carcinomas of lung, breast, prostate, pancreas, and colon, whereas NRP2 is frequently present in non-carcinoma cell lines derived from melanoma, leukaemia, and neuroblastoma (Bielenberg *et al*, 2006). Studies show that overexpression of NRP1 promotes, while blockade of NRP1 inhibits, tumour cell survival and migration (Miao *et al*, 2000; Bachelder *et al*, 2001, 2003; Barr *et al*, 2005; Chabbert-de Ponnat *et al*, 2006), consistent with a pro-tumorigenic role of NRP1 and direct contribution to tumour progression. While some studies point to a direct role of NRP1 in tumour cell functions, a recent report showed that the ability of blocking NRP1 antibodies from inhibiting tumour growth *in vivo* was not dependent on the tumour cell expression of NRP1, or the direct anti-tumour effects of NRP1 blockade, but was mediated by an antiangiogenic effect on the tumour vasculature (Pan *et al*, 2007). It is therefore unclear whether NRP1 is important for tumour cell functions relevant for neoplastic growth and metastatic spread.

We previously characterised a bicyclic peptide, EG3287, based on the C-terminal NRP1-binding domain of VEGF, which specifically blocked VEGF binding to NRP1 and inhibited the anti-chemorepulsive effect of VEGF in dorsal root ganglion neuronal explants (Cheng *et al*, 2004) and the biological effects of VEGF in vascular endothelial cells (Jia *et al*, 2006). In the present study, we investigated the effects of EG3287 on the functions of human carcinoma lung A549, kidney ACHN, and prostate DU145 cells expressing NRP1 but lacking VEGFR-2, and the mechanisms underlying these effects.

## Materials and methods

### Reagents

Recombinant human VEGF (VEGF-A_165_) was obtained from R&D Systems (Abingdon, UK). Antibodies against NRP1, NRP2, VEGFR-1, and VEGFR-2 were purchased from Santa Cruz Biotechnology Inc. (Heidelberg, Germany). A functional blocking antibody against the integrin *β*1-subunit was from Millipore (Livingston, UK). EG3287 (purity >90%) was synthesised by Bachem Inc. (Merseyside, UK) as described previously (Cheng *et al*, 2004; Jia *et al*, 2006). Collagen type-I, cell dissociation solution, Dulbecco's modified Eagle's medium (DMEM)/25 mM HEPES, 5-fluorouracil (5-FU), paclitaxel, and cisplatin were purchased from Sigma-Aldrich (Dorset, UK). All other reagents used were of the purest grade available.

### Cell culture

The human carcinoma cell lines lung A549 and kidney ACHN, originally from ECACC, were provided by Quintiles Limited (Edinburgh, UK) and grown in RPMI-1640 medium/L-glutamine (Invitrogen, Paisley, UK) containing 10% FBS. The human carcinoma cell lines breast MDA-MB-453 and prostate DU145 were purchased from ATCC (Manassas, VA, USA) and cultured in DMEM (Invitrogen, Paisley, UK) containing 10% FBS and RPMI-1640 medium/L-glutamine containing 10% FBS, respectively. Human umbilical vein endothelial cells (HUVECs) were obtained from TCS CellWorks (Buckingham, UK) and cultured in EBM (Cambrex BioScience Ltd, Nottingham, UK) supplemented with human epidermal growth factor, bovine brain extract, and 10% FBS.

### Immunoblotting

Cells were extracted using lysis buffer (64 mM Tris–HCl (pH 6.8), 0.2 mM Na_3_VO_4_, 2% SDS, 10% glycerol, protease inhibitors for serine, cysteine, metalloproteases). The whole-cell lysate samples were separated by SDS–PAGE and transferred to Immobilon membranes (Millipore). The membranes were immunoblotted with specific primary antibodies. Immunoreactive bands were visualised by chemiluminescence using horseradish peroxidase-conjugated secondary antibodies and enhanced chemiluminescence reagent (Amersham Biosciences, Bucks, UK).

### ^125^I-VEGF binding

Binding displacement experiments were performed as described previously (Jia *et al*, 2006), using carcinoma cells grown to confluence in 24-well plates and using the indicted concentrations of EG3287 and 0.1 nM of ^125^I-VEGF-A_165_ (1200–1800 Ci mmol^−1^; GE Healthcare, Little Chalfont, Buckinghamshire, UK). Non-specific binding was determined in the presence of 100-fold excess unlabelled VEGF.

### Cell migration

Cell migration was measured in chemotaxis 24-transwell plates using collagen-I-coated inserts incorporating polyethylene terephthalate track-etched membranes with 8-*μ*m pores (Becton Dickinson Biosciences, Le Pont De Claix, France). Different concentrations of serum or VEGF in RPMI-1640/0.1% BSA were placed in the bottom wells of the plates. Cells were trypsinised, washed, and resuspended in RPMI-1640/0.1% BSA. A total of 1.5 × 10^6^ cells with or without EG3287 treatment, as indicated, were loaded into each top inserts. The chemotaxis transwell plates were incubated at 37°C for 4 h. After the incubation, non-migrated cells on the top side of the transwell membranes were removed, and migrated cells on the underside of the transwell membranes were stained using the REASTAIN Quick-Diff kit (Reagena Ltd, Toivala, Finland). The stained cells from each well were counted in four fields at × 100 magnification using an eyepiece-indexed graticule (100 grids).

### Cell proliferation

Carcinoma cells were seeded at a density of 2 × 10^4^ cells per well of 24-well plates or at the indicated densities in 0.5 ml of RPMI-1640 medium containing 0.5% serum. Five hours after plating, the medium was replaced with fresh medium containing 5% serum, 25 ng ml^−1^ VEGF, or various concentrations of 5-FU, as indicated, in the absence or presence of EG3287 at 100 *μ*M. After 3 days, the cell numbers were determined using a Sysmex CDA-500 cell counter.

### Measurement of VEGF

The concentrations of VEGF (VEGF-A_165_) were determined in the conditioned media of cell culture using a specific immunoassay kit (R&D Systems) according to the manufacturer's instructions.

### RNA interference

A 21-mer annealed small interference RNA (siRNA) targeting human VEGF was obtained from Santa Cruz Biotechnology Inc. Carcinoma cells were transfected with the VEGF siRNA (sc-29520) at 10 nM using the transfection reagent INTERFERin (Polyplus-transfection Inc., Illkirch, France) according to the manufacturer's instructions. Pre-designed 21-mer annealed siRNAs targeting human NRP1 or NRP2 were purchased from Ambion Europe Limited (Huntingdon, UK). Carcinoma cells were transfected with NRP1 siRNA (ID no. 4820), NRP1 siRNA (ID no. 4914), or NRP2 siRNA (ID no. 107264) at 100 nM using the transfection reagent siPORT NeoFX according to the manufacturer's instructions. In parallel, Silencer-negative control #1 siRNA (ID no. 4611), which is a non-targeting scrambled siRNA, and Silencer GAPDH siRNA control (ID no. 4605) were used at the same concentration. The knockdown effects of VEGF, NRP1, or NRP2 siRNA were then determined by real-time quantitative PCR, immunoassay, immunoblotting, and migration assays. Total RNA from transfected cell was extracted using RNeasy kit (Qiagen Ltd, Crawley, UK) and treated with DNase-I. Single-stranded cDNA was reverse-transcribed from total RNA with oligo-d(T)_16_ primer using GeneAmp kit (Applied Biosystems, Austin, TX, USA. Primers (synthesised by Sigma Genosys, Gillingham, UK) for real-time PCR were designed to flank the 3′-untranslated region of the gene sequence to ensure the specificity of the fragment, and to ensure that amplified fragments were 200–250 base pairs (Supplementary Table 1). Amplification of predicted fragments was verified by conventional RT-PCR. Real-time quantitative PCR was performed using the LightCycler-FastStart DNA Master SYBR Green I kit and the Lightcycler System (Roche Diagnostics, Lewes, UK), as previously described (Jia *et al*, 2004).

### Cell–matrix adhesion

Cell adhesion to extracellular matrix (ECM) proteins, including basement membrane protein complex (BMC), laminin-I, collagen-IV, or fibronectin, was measured by the Innocyte ECM cell adhesion assay (Calbiochem Inc., Nottingham, UK). Cells were detached with a non-enzyme cell dissociation solution, washed, and resuspended in RPMI-1640 medium. Cells were pretreated with EG3287 or the function-blocking integrin-*β*1 antibody at various concentrations as indicated for 30 min and then seeded at a density of 3 × 10^4^ cells per matrix-coated well in 96-well plates. After 1.5 h of incubation the cells were washed with PBS. The attached cells were labelled with the green fluorescent dye calcein-AM and measured using a fluorescence plate reader at an excitation wavelength of 485 nm and an emission wavelength of 510 nm.

### Cell viability

Cell viability was determined by measuring the conversion of the tetrazolium salt XTT to form formazan dye. Carcinoma cells were seeded at a density of 4 × 10^3^ cells per well on non-coated or fibronectin-coated 96-well plates in 100 *μ*l serum-free medium containing various concentrations of 5-FU, paclitaxel, or cisplatin as indicated in the absence or presence of EG3287 at 100 *μ*M. After 44 h of incubation, the XTT labelling reagent mixture (Roche Diagnostics, East Sussex, UK) was added to the cultures and they were incubated for a further 4 h. The formazan product was then measured at A_490 nm_ with a reference wavelength at 595 nm.

### Statistical analysis

Data were analysed using Prism (version 4.0) statistical packages. Comparisons of two sets of continuous variables were performed using Student's *t*-test or *t*-test with Welch's correction where appropriate. Differences among three or four concentrations of compounds were evaluated by the one-way analysis of variance (ANOVA) with Bonferroni's multiple comparison tests. Differences between two treatment groups at various concentrations were analysed using the two-way ANOVA with Bonferroni's post-tests. The values represent means±s.e.m. determined from the results of three independent experiments each performed in duplicates or triplicates unless where stated. *P*<0.05 was considered statistically significant.

## Results

### Inhibition of ^125^I-VEGF binding and VEGF-induced migration in NRP1-expressing lung carcinoma cells by EG3287

**Figure 1:**
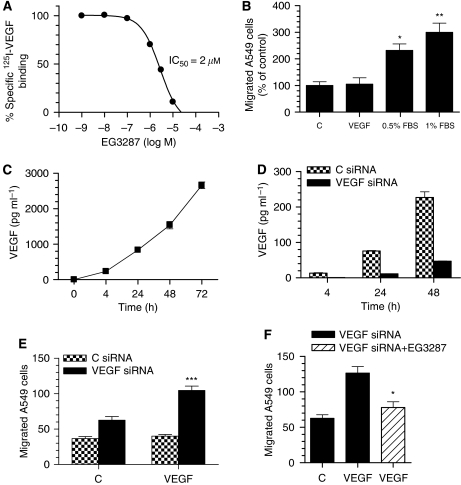
Inhibition of ^125^I-VEGF binding to NRP1-expressing carcinoma cells by EG3287 and the effects of VEGF on cell migration. (**A**) Confluent A549 cells were incubated for 2 h at 4°C with 0.1 nM ^125^I-VEGF in the presence of the indicated concentrations of EG3287. (**B**) Carcinoma cells were placed into the top inserts and either no addition (control, C), 25 ng ml^−1^ VEGF, or 0.5 or 1% FBS was added to the bottom wells of the transwell plates. Chemotaxis of these cells towards VEGF or serum was determined after 4 h of incubation. ^*^P<0.05; ^**^P<0.01 *vs* control. (**C**) Vascular endothelial growth factor was measured by ELISA in the conditioned medium collected from cultured A549 cells at the indicated time points after incubation of cells in serum-free medium. Values represent means±s.d. of VEGF (pg ml^−1^). (**D**) A549 cells were transfected with control (C) or VEGF siRNA and incubated in serum-free medium for the time periods indicated, after which VEGF was measured in the conditioned medium. (**E**) A549 cells transfected with control or VEGF siRNA were placed into the top inserts, either no addition (C) or 25 ng ml^−1^ VEGF was added to the bottom wells of the transwell plates and chemotaxis towards VEGF was determined after 4 h of incubation. ^***^P<0.001 *vs* control siRNA. (**F**) VEGF siRNA-transfected cells were pretreated for 30 min with EG3287 at 100 *μ*M and chemotaxis towards 25 ng ml^−1^ VEGF was determined after 4 h of incubation. ^*^P<0.05 *vs* VEGF siRNA without EG3287.

The NRP1 antagonist EG3287 was first tested for its ability to compete VEGF binding to NRP1-expressing lung carcinoma A549 cells (Supplementary Figure 1). EG3287 displaced the specific binding of ^125^I-VEGF to A549 cells with a half-maximal inhibition (IC_50_) of 2 *μ*M (Figure 1A). Inhibition of ^125^I-VEGF binding by the peptide was concentration-dependent and reached a maximum of 100% inhibition at 24 *μ*M. The potency and efficacy of the peptide were very similar to previous results from porcine aortic endothelial cells expressing NRP1 and human breast carcinoma MDA-MB-231 cells (Jia *et al*, 2006).

Since NRP1 has been strongly implicated in the migratory response to VEGF in endothelial cells (Soker *et al*, 1998; Whitaker *et al*, 2001; Bernatchez *et al*, 2002; Lee *et al*, 2002), initially we examined the effects of EG3287 on tumour cell chemotaxis induced by VEGF. While A549 cells expressed levels of NRP1 and NRP2 similar to those in endothelial cells, western blotting showed that A549 cells had no detectable protein expression of the main signalling receptor for VEGF, VEGFR-2 (Supplementary Figure 1). Exogenous VEGF at 25 ng ml^−1^ did not increase the migration of A549 cells through a collagen-coated membrane after 4 h of incubation using a 24-transwell chamber assay (Figure 1B). We also evaluated whether exogenous VEGF could stimulate carcinoma cell proliferation. There was no effect on A549 cell proliferation as determined by counting cell numbers after a 3-day incubation with VEGF relative to untreated control (Supplementary Figure 2A). However, carcinoma cells responded well to serum stimulation in assays of cell migration and growth (Figure 1B and Supplementary Figure 2A).

Determination of endogenously produced VEGF (VEGF-A_165_) levels in the conditioned media of A549 cells by ELISA showed a significant increase in VEGF after 4 h (235 pg ml^−1^) and higher levels after 48 h (1.5 ng ml^−1^) and 72 h (2.7 ng ml^−1^) of incubation (Figure 1C). These results suggested that the effects of exogenously added VEGF might be masked by secretion of endogenous VEGF. This was investigated by examining the effect of endogenous VEGF knockdown using a specific siRNA. As shown in Figure 1D, siRNA specifically targeted at VEGF markedly reduced VEGF production as measured by ELISA using cell culture supernatants after different time periods of cell incubation. We then determined whether siRNA-mediated endogenous VEGF knockdown modulated the migratory response of tumour cells to exogenous VEGF. A549 cells in which VEGF gene expression had been inhibited by siRNA, exhibited increased migration in response to a gradient of exogenous VEGF (Figure 1E). This enhanced migratory response to exogenous VEGF was blocked by pretreatment with the NRP1 antagonist, EG3287 (Figure 1F).

**Figure 2:**
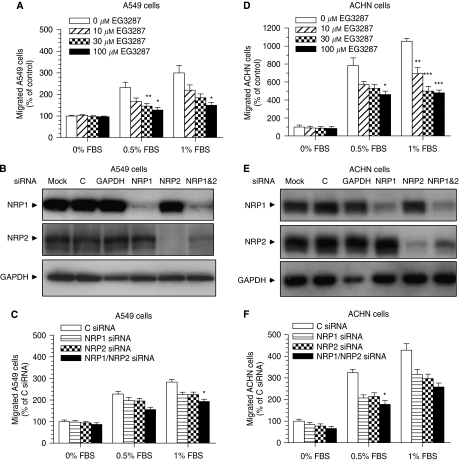
Inhibition of migration of carcinoma cells by EG3287 and NRP1 silencing. (**A**, **D**) A549 and ACHN cells were pretreated for 30 min with the indicated concentrations of EG3287 and chemotaxis towards 0, 0.5, or 1% FBS was determined after 4 h of incubation. ^*^P<0.05; ^**^P<0.01; and ^***^P<0.001 *vs* untreated control. (**B**, **E**) A549 and ACHN cells were transfected with NeoFX only as a mock control (M) or siRNAs, as indicated, and total cellular protein was extracted 48 h after transfection, and NRP1 and NRP2 were detected by immunobloting. GAPDH served as loading control. (**C**, **F**) siRNA-transfected A549 and ACHN cells were loaded into the top inserts of transwell plates and chemotaxis towards 0, 0.5, or 1% FBS was determined after 4 h of incubation. ^*^P<0.05 *vs* control siRNA.

We also studied the effects of EG3287 on carcinoma cell migration in response to serum chemoattraction. Treatment of A549 cells with EG3287 at various concentrations effectively reduced serum-induced cell migration in a concentration-dependent manner (Figure 2A). Compared with untreated control cells, EG3287 almost abolished the increased cell migration induced by serum.

To further investigate the role of NRP1 in carcinoma cell migration, we used RNA interference to knock down NRP1 expression. Transfection of A549 cells with two individual specific siRNAs targeted against NRP1 mRNA showed effective knockdown of NRP1 gene expression (by 82% Supplementary Figure 2B) and protein expression (Figure 2B and Supplementary Figure 2C), as compared with control siRNA samples. Lowering NRP1 expression by both NRP1 siRNAs inhibited the migration of A549 cells by 21 and 31% as compared with that in control siRNA-transfected cells (Figure 2C and Supplementary Figure 2D). Specific NRP2-targeted siRNA also decreased the chemotaxis of A549 cells to a similar extent (Figure 2B and C). Double knockdown of NRP1 and NRP2 caused greater inhibition of the migration of A549 cells as compared with single knockdown.

Similar results were found using human kidney carcinoma ACHN cells, which also express NRP1 but not VEGFR-2 (Supplementary Figure 1). EG3287 potently competed ^125^I-VEGF binding to ACHN cells with the same IC_50_ of 2 *μ*M (Supplementary Figure 3A). The treatment with EG3287 caused dose-dependent inhibition of ACHN cell chemotaxis towards serum, with a maximum effect of >50% inhibition (Figure 2D). The migration of ACHN cells was also reduced by siRNA-mediated knockdown of either NRP1 or NRP2 gene and protein expression (Supplementary Figure 3B and Figure 2E and F). We verified that NRP1 siRNA transfection did not affect NRP2 expression and NRP2 siRNA transfection did not silence NRP1 expression in both lung A549 and kidney ACHN carcinoma cells (Figure 2B and E).

### EG3287 suppressed carcinoma cell adhesion to ECM proteins

**Figure 3:**
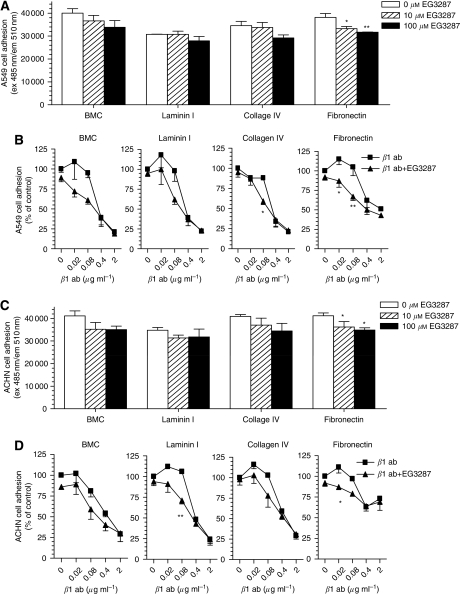
Suppression of cell–matrix adhesion and enhancement of the inhibitory effects of an integrin-*β*1-function-blocking antibody by EG3287. (**A**, **C**) A549 and ACHN cells were pretreated for 30 min with the indicated concentrations of EG3287 and seeded to wells coated with the indicated matrix proteins. After 90 min of incubation, attached cells were labelled with calcein-AM and measured using a fluorescence plate reader. ^*^P<0.05; ^**^P<0.01 *vs* untreated control. (**B**, **D**) A549 and ACHN cells were pretreated for 30 min with an integrin-*β*1-blocking antibody at the indicated concentrations in the absence or presence of 100 *μ*M EG3287, and cell adhesion to matrix proteins was measured. ^*^P<0.05; ^**^P<0.01 for integrin-*β*1 antibody alone *vs* integrin-*β*1 antibody plus EG3287.

We next evaluated the effects of EG3287 on cell adhesion to ECM proteins, an important step in cancer cell spread, migration, and invasion. The adhesion of carcinoma cells was determined by measuring adhesion to the ECM proteins, BMC, laminin-I, collagen-IV, and fibronectin. As illustrated in Figure 3A, EG3287 treatment generally decreased the adhesion of lung carcinoma A549 cells to ECM proteins. Compared with untreated controls, EG3287 caused a significant and dose-dependent inhibition of A549 cell adhesion to fibronectin.

Since the integrin *β*1-subunit mediates specific cell binding to laminin, collagen, and fibronectin, and has been shown to be a major integrin receptor expressed in carcinoma cells, we evaluated whether there was possible cooperation of NRP1 with integrin-*β*1 in carcinoma cell–matrix adhesion. Figure 3B showed that disruption of integrin-*β*1 ligation using a function-blocking integrin-*β*1 antibody at concentrations of 0.4–2 *μ*g ml^−1^ markedly reduced the adhesion of A549 cells to matrix proteins, but had little effect at lower concentrations. In the presence of EG3287, the inhibition of A549 cell adhesion to collagen-IV and fibronectin was significantly potentiated with the integrin antibody at concentrations of 16 and 80 ng ml^−1^.

In renal carcinoma ACHN cells, similar inhibitory effects of EG3287 on matrix adhesion were observed (Figure 3C). EG3287 also significantly potentiated the inhibition of ACHN cell adhesion to laminin-I and fibronectin by the integrin antibody at lower concentrations (Figure 3D).

### Effects of EG3287 on cell growth, survival, and response to chemotherapeutic agents

**Figure 4:**
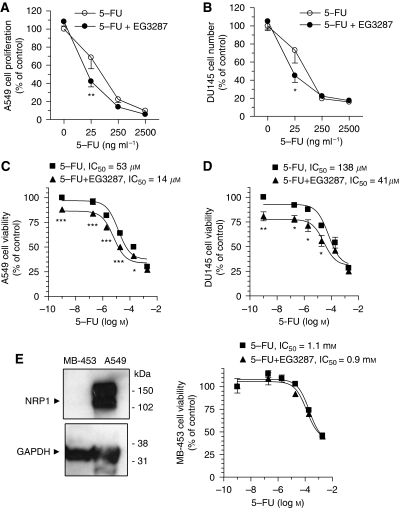

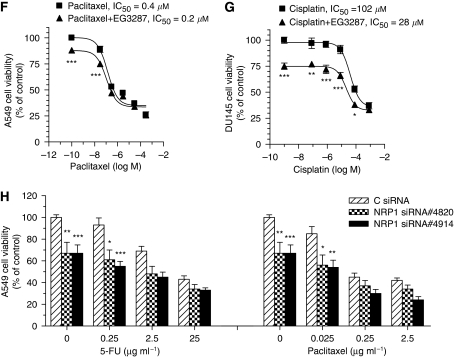
Sensitisation of carcinoma cells to chemotherapeutic agents by EG3287. (**A**, **B**) A549 and DU145 cells were incubated in medium containing 5% serum with 5-FU at the indicated concentrations in the absence or presence of 100 *μ*M EG3287. Cell numbers were determined after a 3-day incubation. ^*^P<0.05; ^**^P<0.01 for 5-FU alone *vs* 5-FU plus EG3287. (**C**, **D**) A549 and DU145 cells were incubated in serum-free medium containing 5-FU at the indicated concentrations in the absence or presence of 100 *μ*M EG3287. Cell viability was measured after 48 h of treatment. ^*^P<0.05; ^**^P<0.01; and ^***^P<0.001 for the chemotherapeutic drug alone *vs* drug plus EG3287. (**E**) Left, total cellular proteins were extracted from breast carcinoma MDA-MB-453 and lung carcinoma A549 cells and NRP1 was detected by immunobloting. *Right:* MDA-MB-453 cells were incubated in serum-free medium containing 5-FU at the indicated concentrations in the absence or presence of 100 *μ*M EG3287. Cell viability was measured after 48 h of treatment. (**F**, **G**) A549 and DU145 cells were incubated in serum-free medium containing paclitaxel or cisplatin at the indicated concentrations in the absence or presence of 100 *μ*M EG3287. Cell viability was measured after 48 h of treatment. ^*^P<0.05; ^**^P<0.01; and ^***^P<0.001 for the chemotherapeutic drug alone *vs* drug plus EG3287. (**H**) Small interference RNA-transfected A549 cells were incubated in serum-free medium containing 5-FU or paclitaxel at the indicated concentrations for 48 h prior to viability measurement. ^*^P<0.05; ^**^P<0.01; and ^***^P<0.001 *vs* control (C) siRNA-transfected cells.

The anticancer potential of the NRP1 antagonist was further investigated by determining the effects of NRP1 antagonism on the response of carcinoma cells to a chemotherapeutic agent, 5-fluouracil (5-FU), which possesses a broad spectrum of therapeutic activity against various cancers, including non-small-cell lung cancer. Treatment of lung carcinoma A549 cells with 5-FU for 3 days caused a dose-dependent inhibition of cell proliferation (Figure 4A). As compared with untreated controls, treatment of A549 cells with 5-FU at 25 ng ml^−1^ suppressed cell proliferation by 32%. Treatment with 25 ng ml^−1^ 5-FU in combination with 100 *μ*M EG3287 caused a further significant reduction (58%) in the number of proliferating cells (*P*<0.01 *vs* 5-FU alone). However, kidney carcinoma ACHN cells responded poorly to treatment with 5-FU at lower concentrations and the combined treatment with 5-FU and EG3287 had no greater effect on cell proliferation compared with the single agent 5-FU (Supplementary Figure 3C). Human prostate carcinoma DU145 cells, which express NRP1 but not VEGFR-2 (Supplementary Figure 1), were then used to examine cell growth in response to chemotherapy. Similar to A549 cells, the inhibitory effects on the proliferation of DU145 cells were enhanced by treatment with 5-FU and EG3287 together (Figure 4B). EG3287 alone had no inhibitory effects on cell proliferation stimulated by serum.

The effect of EG3287 on 5-FU cytotoxicity was next assessed using the XTT assay of carcinoma cell viability. Treatment with 5-FU for 2 days induced a decrease in the viability of lung carcinoma A549 cells in a concentration-dependent manner, with an IC_50_ of 53 *μ*M (Figure 4C). In the presence of EG3287, 5-FU showed a >3-fold increase in its potency in reducing cell viability, with an IC_50_ of 14 *μ*M. In DU145 cells, 5-FU combined with EG3287 showed a similar (>3-fold) increase in cytotoxic potency (Figure 4D), although DU145 cells responded to the drug with an IC_50_ of 138 *μ*M, compared with 53 *μ*M in A549 cells. Interestingly, EG3287 alone at 100 *μ*M modestly reduced the survival of both lung A549 and prostate DU145 carcinoma cells in the absence of serum. In contrast, EG3287 caused no alterations in the cytotoxic effects of 5-FU in breast carcinoma MDA-MB-453 cells, which are NRP1-negative (Figure 4E).

Further studies were performed using paclitaxel and cisplatin, different classes of chemotherapeutic agents clinically used as frontline treatment for advanced non-small-cell lung cancer and prostate cancer, respectively. As shown in Figure 4F, the cytotoxic activity of paclitaxel on lung carcinoma A549 cells was dose-dependent and exhibited greater potency than 5-FU. The combination of paclitaxel and EG3287 caused a further decrease in cell viability (IC_50_=0.2 *μ*M) as compared with treatment with paclitaxel alone (IC_50_=0.4 *μ*M). Consistently, EG3287 increased the cytotoxic activity of cisplatin in prostate carcinoma DU145 cells with an improved IC_50_ of 28 *μ*M (Figure 4G). The involvement of NRP1 in carcinoma cell survival and drug response was also examined by downregulation of NRP1 expression. In agreement with NRP1 antagonism, NRP1 silencing with two individual specific siRNAs in lung carcinoma A549 cells decreased cell viability and increased the cytotoxic effects of both 5-FU and paclitaxel as compared with that in control siRNA-transfected cells (Figure 4H).

### EG3287 decreased fibronectin-mediated cell viability and chemoresistance

**Figure 5:**
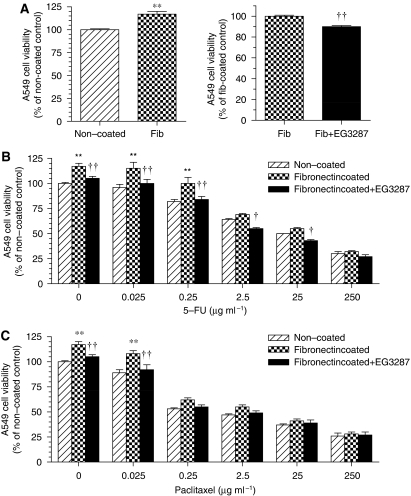
Effects of EG3287 on A549 cell survival in the presence of chemotherapeutic drugs and fibronectin. (**A**) A549 cells were seeded on non-coated or fibronectin (Fib)-coated 96-well plates in serum-free medium in the absence or presence of 100 *μ*M EG3287 and cell viability was measured after 48 h of treatment. ^**^P<0.01 *vs* non-coated control; ^††^*P*<0.01 *vs* untreated control. (**B**, **C**) A549 cells were seeded on non-coated or fibronectin-coated 96-well plates in serum-free medium containing 5-FU or paclitaxel at the indicated concentrations in the absence or presence of 100 *μ*M EG3287. ^**^P<0.01 *vs* non-coated control; ^†^*P*<0.05; ^††^*P*<0.01 for 5-FU or paclitaxel plus EG3287 *vs* 5-FU or paclitaxel alone on fibronectin.

Since adhesion of cancer cells to ECM is associated with increased resistance to several cytotoxic drugs (Broxterman *et al*, 2003), we next examined the response to the chemotherapeutic drug and EG3287 of A549 cells grown on either fibronectin-coated or non-coated plates. As shown in Figure 5A, the presence of fibronectin increased the number of viable cells as compared with non-coated controls. Fibronectin also increased cell viability in the presence of 5-FU or paclitaxel at concentrations of 0.025–0.25 *μ*g ml^−1^, as compared with uncoated controls (Figure 5B and C). However, in the presence of fibronectin, the NRP1 antagonist EG3287 significantly decreased cell viability (Figure 5A), and fibronectin-dependent cell survival in the presence of 5-FU or paclitaxel was abolished by EG3287 treatment (Figure 5B and C).

**Figure 6:**
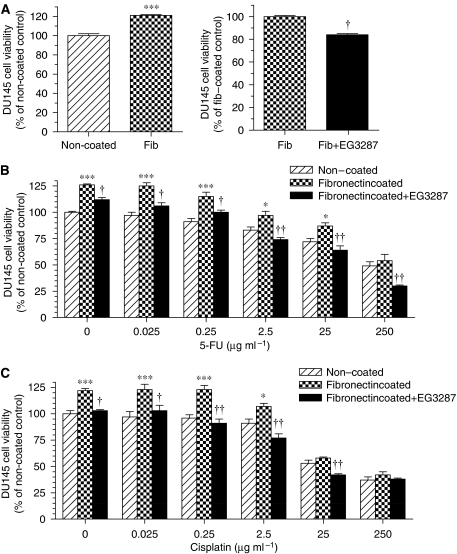
Effects of EG3287 on DU145 cell survival in the presence of chemotherapeutic drugs and fibronectin. (**A**) DU145 cells were seeded on non-coated or fibronectin (Fib)-coated 96-well plates in serum-free medium in the absence or presence of 100 *μ*M EG3287 and cell viability was measured after 48 h of treatment. ^***^P<0.001 *vs* non-coated control; ^†^*P*<0.05 *vs* untreated control. (**B**, **C**) DU145 cells were seeded on non-coated or fibronectin-coated 96-well plates in serum-free medium containing 5-FU or cisplatin at the indicated concentrations in the absence or presence of 100 *μ*M EG3287. ^*^P<0.05; ^***^P<0.001 *vs* non-coated control; ^†^*P*<0.05; ^††^*P*<0.01 for 5-FU or cisplatin plus EG3287 *vs* 5-FU or cisplatin alone on fibronectin.

In prostate carcinoma DU145 cells, a similar increase in cell viability was found in the presence of fibronectin as compared with non-coated controls (Figure 6A). The cytotoxic effects of 5-FU or cisplatin at concentrations of 0.025–2.5 *μ*g ml^−1^ were also reduced in the presence of fibronectin (Figure 6B and C). Treatment of DU145 cells with EG3287 significantly prevented fibronectin-mediated cell survival (Figure 6A) and blocked the fibronectin-dependent cell resistance to 5-FU or cisplatin (Figure 6B and C).

## Discussion

In the present study, we investigated the effects of the NRP1 antagonist EG3287 on cell proliferation, survival, migration, and adhesion to matrix in the NRP1-expressing carcinoma cell lines, non-small-cell lung A549, kidney ACHN, and prostate DU145 cells. A major conclusion of this study is that the NRP1 antagonist EG3287 markedly inhibits the chemotactic migration of carcinoma lung A549 and kidney ACHN cells. Other studies have examined the roles of NRP1 in tumour cell survival and proliferation (Bachelder *et al*, 2001; Barr *et al*, 2005; Chabbert-de Ponnat *et al*, 2006), but the role of NRP1 in tumour cell migration is less well understood. In the present study, we have shown that the NRP1 antagonist EG3287 significantly inhibited the migration of NRP1-positive lung carcinoma A549 cells in response to VEGF and serum. Small interference RNA-mediated inhibition of NRP1 expression also reduced the migration of both A549 and ACHN cells. Since migration of tumour cells plays a key role in neoplastic spread, invasion of surrounding tissue, and formation of metastasis, these findings indicate a key role for NRP1 in the motility of carcinoma cells, which may contribute to tumour progression and metastatic potential.

Our results indicate that an important mechanism through which NRP1 antagonism may inhibit tumour cell migration is by reducing cell adhesion to ECM. Neuropilin-1 functioned as a cell–cell adhesion molecule when overexpressed in a mouse fibroblast cell line (Takagi *et al*, 1995), and the b1 and b2 domains of the NRP1 extracellular domain are essential for cell aggregation activity independent of VEGF or semaphorin ligands (Shimizu *et al*, 2000). However, the cell–matrix adhesive properties of NRP1 have not been investigated previously in carcinoma cells expressing endogenous NRP1. EG3287 reduced the adhesion of both lung A549 and kidney ACHN carcinoma cells to ECM, suggesting an important role of NRP1 as a regulator, at least in part, of carcinoma cell attachment to ECM. Furthermore, NRP1 antagonism enhanced the inhibitory effect of function-blocking integrin-*β*1 antibody on carcinoma cell adhesion to ECM, indicating that NRP1 synergistically cooperates with integrin-*β*1 to promote carcinoma cell adhesion to matrix proteins. Since EG3287 had no significant effect on the cell expression of integrin-*β*1 (data not shown), the inhibitory effects of the NRP1 antagonist on carcinoma cell responses, including adhesion and migration, were unlikely to have been mediated through downregulation of integrin-*β*1 expression. Fukasawa *et al* (2007) recently reported an association between NRP1 and integrin-*β*1 subunit in PANC-1 pancreatic cancer cells. Our data are the first to show a functional interaction of NRP1 with integrin-*β*1 in cell–matrix adhesion. Such an interaction may contribute to integrin and growth factor receptor crosstalk found in some cell types (Byzova *et al*, 2000; Eliceiri, 2001; Streuli and Akhtar, 2009). For example, stimulation of proliferation of epithelial cells and fibroblasts with epidermal growth factor depends on *β*1-integrins, whereas VEGF-promoted adhesion and migration of endothelial cells are mediated via integrin-*β*1, *α*v*β*3, and *α*v*β*5. Our findings implicate a role for NRP1 in promoting integrin-mediated cancer cell attachment and migration into ECM.

A549 and ACHN carcinoma cells also expressed NRP2 at a level similar to that of NRP1. Furthermore, NRP2 siRNA also decreased tumour cell migration, and combined transfection with NRP1 and NRP2 siRNAs had a greater effect. While a role for NRP1 in VEGF-dependent cell migration in endothelial cells is well-established, the role of NRP2 in cell migration has previously not been much investigated, although VEGF-A_165_ is known to bind with high affinity to NRP2. Our results indicate that NRP2 plays a role in the migration of A549 and ACHN carcinoma cells, and further studies aimed at investigating the possible cooperation and interaction between NRP1 and NRP2 would be of interest.

Another important finding of our study is that the NRP1 antagonist sensitised carcinoma cells to the clinically important chemotherapeutic agents 5-FU, paclitaxel, and cisplatin. The results showed that EG3287-treated lung A549 and prostate DU145 cells were more susceptible to the cytotoxic effects of 5-FU, paclitaxel, and cisplatin at suboptimal concentrations as compared with the chemotherapeutic agents administered alone. Similarly, downregulation of NRP1 by siRNAs in A549 cells sensitised the cell response to the chemotherapeutic agents. The notion that NRP1 may play a role in mediating chemoresistance is supported by the finding that overexpression of NRP1 promotes chemoresistance in human FG pancreatic cancer cells (Wey *et al*, 2005). Our finding that EG3287 combined with chemotherapy in lung A549 and prostate DU145 cells prevented fibronectin-dependent chemoresistance, indicates that the mechanism underlying NRP1-mediated chemoresistance is mediated in part through the integrin-dependent interaction of carcinoma cells with the ECM. Interestingly, it has shown that adhesion of leukaemia cells to fibronectin via integrin-*β*1 contributes to cell adhesion-mediated drug resistance (Hazlehurst *et al*, 2007). Although we found a modest anti-survival effect of EG3287 on carcinoma A549 and DU145 cells in serum-free medium, we were unable to observe any growth-inhibitory effects of EG3287 alone on carcinoma cells in response to serum stimulation, which suggests that NRP1 plays a less important role in proliferation of these cells. Consistent with our observations, overexpression of NRP1 increased motility in colon and prostate carcinoma cells, but had no effect on cell mitogenesis and proliferation (Miao *et al*, 2000; Ochiumi *et al*, 2006).

Several previous investigations of the role of NRP1 in tumour cells have shown NRP1 as a functional VEGF receptor. Thus, NRP1 mediates the chemotaxis and survival of VEGF autocrine functions in breast carcinoma MDA-MB-231 cells lacking VEGFR-2 (Bachelder *et al*, 2001, 2003), and in Dunning rat prostate carcinoma AT2.1 cells, which do not express VEGFR-2, NRP1 overexpression increases cell migration and reduces cell apoptosis *in vivo* (Miao *et al*, 2000). Since lung carcinoma A549 cells expressed no detectable VEGFR-2, the chemotactic migration of carcinoma cells was highly unlikely to be mediated via VEGFR-2. These and other findings pose the problem of the mechanism through which VEGF acts in tumour cells, since the present study together with previous work shows that NRP1 is expressed in diverse tumour cells in the absence of significant expression of the major signalling VEGF receptor, VEGFR-2 (Soker *et al*, 1998; Bachelder *et al*, 2003; Simiantonaki *et al*, 2008). The possibilities are that NRP1 mediates tumour cell functions either in a VEGF-dependent manner, but independent of VEGFR-2 signalling, or via interaction with other cell-surface receptors and ligands, to transduce signalling and biological functions. A recent paper describing the effects of NRP1 antibodies that specifically block VEGF binding to the b1/b2 domain, concluded that the effects of NRP1 inhibition on endothelial cell function and angiogenesis appeared to be partly independent of VEGF, and also reported no effects of blocking NRP1 antibodies on tumour cell proliferation, but did not examine the effects on tumour cell migration and adhesion (Pan *et al*, 2007). Many tumour cells produce high levels of VEGF, which may block the effects of exogenous VEGF by saturating and/or downregulating surface receptors. In the present paper, while treatment of NRP1-expressing carcinoma cells with exogenous VEGF had no effects on growth and migration, the effects of VEGF on migration in A549 cells were unmasked by siRNA-mediated inhibition of endogenous VEGF production, most likely because endogenous VEGF production limits the formation of a chemoattractant gradient. Overall, we conclude that the inhibitory effects of the NRP1 antagonist, EG3287, on A549 cell migration and adhesion are mediated via a VEGF-dependent but VEGR-2-independent mechanism. Interestingly, the hepatocyte growth factor (HGF) has been identified recently as an additional ligand for NRP1, which potentiated HGF/c-Met signalling and promoted glioma progression and pancreatic cancer cell invasion (Hu *et al*, 2007; Matsushita *et al*, 2007). It is possible, therefore, that NRP1 antagonists and siRNAs could indirectly affect tumour cell function by impairing functional signalling mediated via other receptors such as c-Met. Understanding the molecular basis for the chemotactic effects of VEGF in VEGFR-2-negative tumour cells, and the role played by NRP1, warrant further work.

There is increasing evidence that NRP1 plays a direct role in tumour cell biology and becomes an attractive target for anticancer strategy. The present study suggests that peptide antagonists of NRP1 may be therapeutically useful for preventing tumour cell functions required for metastasis and tumour spread. In addition, the finding that EG3287 sensitises carcinoma cells to paclitaxel, 5-FU, and cisplatin is of interest since most chemotherapeutic agents have limited efficacy and unwanted side effects, and raises the possibility that NRP1 antagonism may have anticancer potential in combination with conventional chemotherapeutics. Theoretically, combinations of conventional chemotherapy with targeted biological therapy for specific patients are especially appealing because such approaches may improve clinical efficacy with minimal adverse events.
