# Supplementary material for: Neuropilin-1 antagonism in human carcinoma cells inhibits migration and enhances chemosensitivity
Source: Br J Cancer. 2010 Jan 19;102(3):541–52. doi: 10.1038/sj.bjc.6605539 (PMC2822953; doi:10.1038/sj.bjc.6605539)
Supplement: Supplementary Figure 1–3 [file 6605539x1.ppt]

## Slide 1
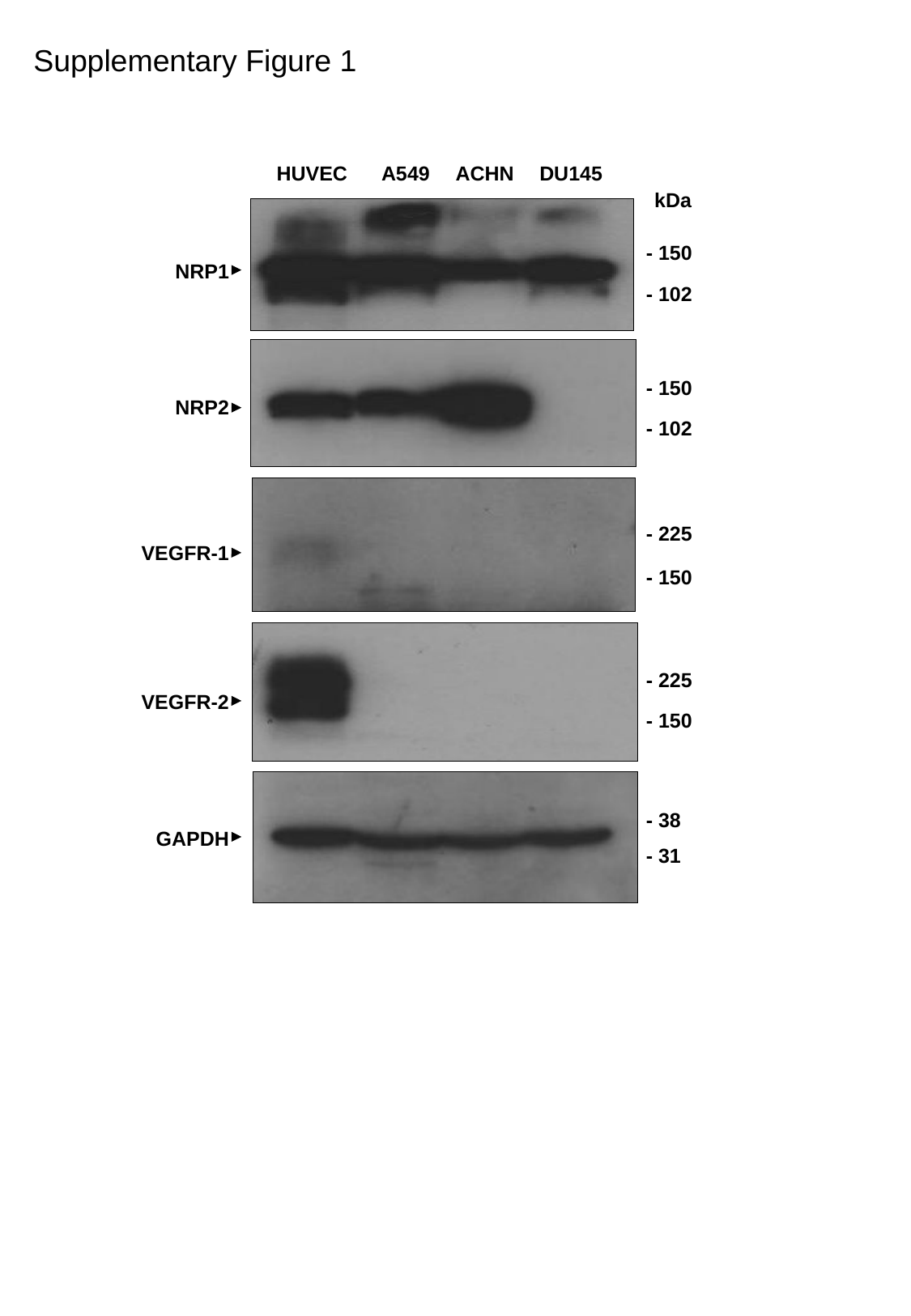

Supplementary Figure 1
#
HUVEC
A549
ACHN
DU145
kDa
- 150
NRP1
- 102
- 150
NRP2
- 102
- 225
VEGFR-1
- 150
- 225
VEGFR-2
- 150
- 38
GAPDH
- 31

## Slide 2
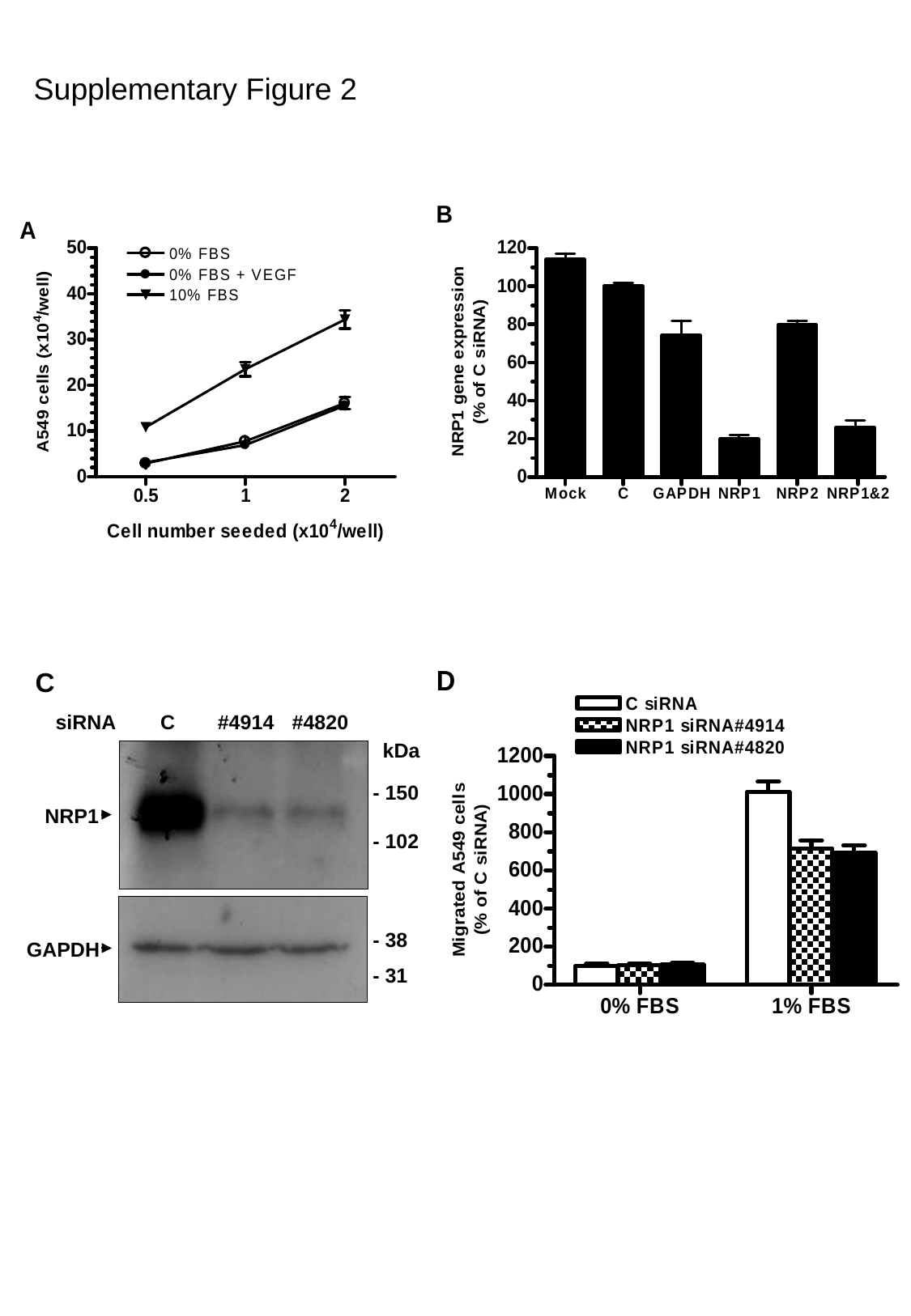

Supplementary Figure 2
#
C
siRNA
C
#4914
#4820
kDa
- 150
NRP1
- 102
- 38
GAPDH
- 31

## Slide 3
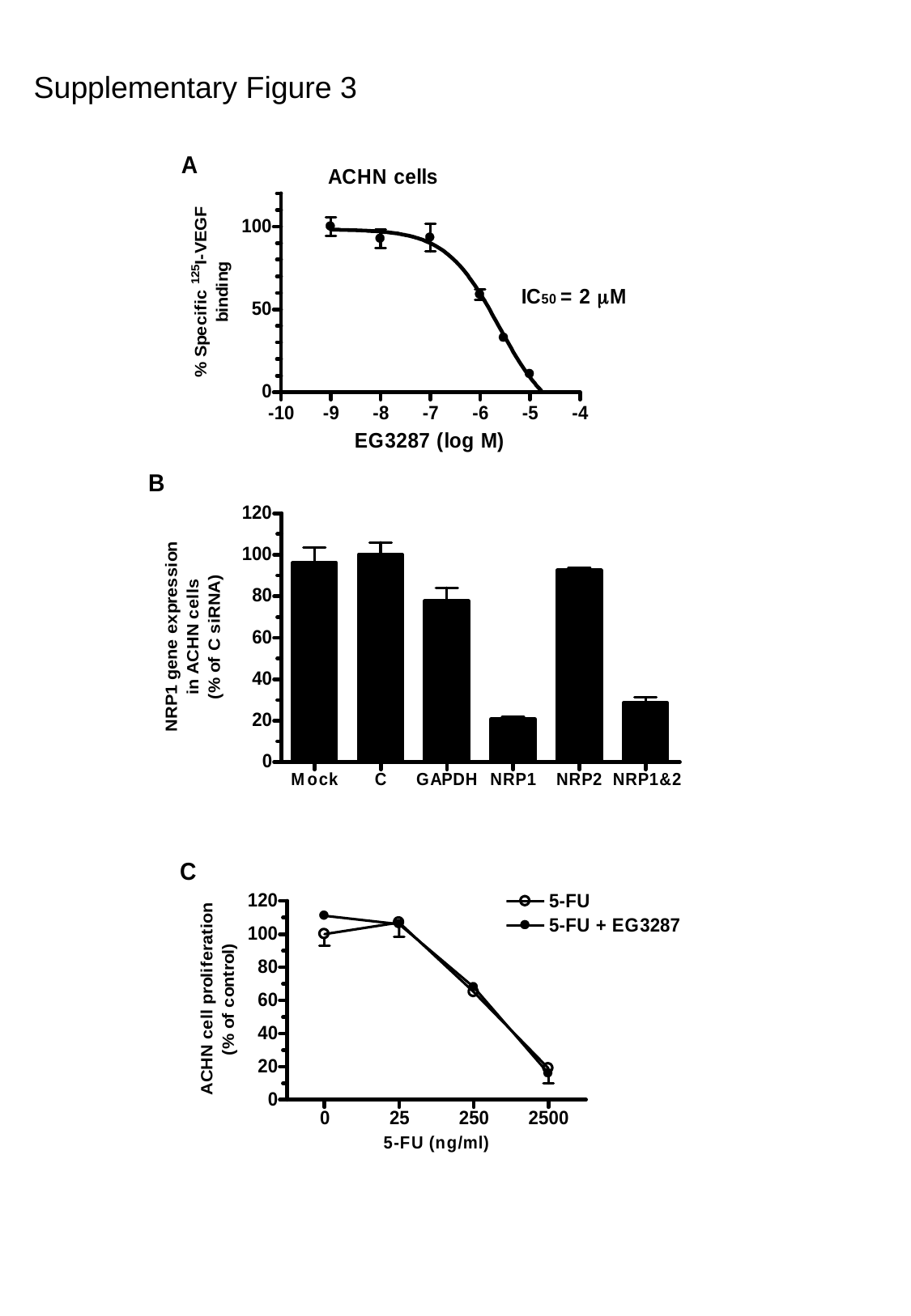

Supplementary Figure 3
#
