# Supplementary material for: Neuropilin-1 antagonism in human carcinoma cells inhibits migration and enhances chemosensitivity
Source: Br J Cancer. 2010 Jan 19;102(3):541–52. doi: 10.1038/sj.bjc.6605539 (PMC2822953; doi:10.1038/sj.bjc.6605539)
Supplement: Supplementary Figure Legends [file 6605539x2.doc]

**Supplementary figure legends**

**Supplementary Figure 1.** Expression of NRP1 and NRP2 in tumour cell lines. Total cellular proteins were extracted from the cell lines as indicated. NRP1, NRP2, VEGFR-1, and VEGFR-2 were detected by immunoblotting. GAPDH served as a loading control. HUVECs served as positive controls, which express NRP1, NRP2, VEGFR-1 and VEGFR-2.

**Supplementary Figure 2.** Effects of VEGF on cell proliferation and siRNA-mediated inhibition of NRP1 expression and cell migration. **(A)** Lung carcinoma A549 cells seeded at various densities as indicated and grew in the absence or presence of 25 ng/ml VEGF or 10% FBS. Cell numbers were determined after 3-day incubation. **(B)** A549 cells were transfected with either NeoFX only as a mock control (M), control siRNA (C), GAPDH siRNA (GAPDH), NRP1 siRNA#4914 (NRP1), NRP2 siRNA (NRP2), or NRP1 siRNA plus NRP2 siRNA (NRP1&2). 48 h after transfection, total RNA was extracted and cDNA was prepared. NRP1 gene expression was detected by real-time quantitative PCR. **(C)** A549 cells were transfected with control siRNA (C), NRP1 siRNA#4914 (#4914), or NRP1 siRNA#4820 (#4820), total cellular protein was extracted 48 h after transfection, and NRP1 was detected by immunobloting. GAPDH served as a loading control. **(D)** siRNA-transfected A549 cells were loaded into top inserts of transwell plates, and chemotaxis towards 0% or 1% FBS was determined after 4 h incubation.

**Supplementary Figure 3.** Inhibition of ^125^I-VEGF binding to NRP1-expressing carcinoma ACHN cells by EG3287, siRNA-mediated inhibition of NRP1 mRNA expression and effects of EG3287 on carcinoma cell chemoresponse. **(A)** Confluent renal carcinoma ACHN cells were incubated for 2 h at 4˚C with 0.1 nM ^125^I-VEGF in the presence of the indicated concentrations of EG3287. **(B)** ACHN cells were transfected with either NeoFX only as a mock control (M), control siRNA (C), GAPDH siRNA (GAPDH), NRP1 siRNA#4914 (NRP1), NRP2 siRNA (NRP2), or NRP1 siRNA plus NRP2 siRNA (NRP1&2). 48 h after transfection, total RNA was extracted and cDNA was prepared. NRP1 gene expression was detected by real-time quantitative PCR. **(C)** ACHN cells were incubated in medium containing 5% serum with 5-FU at the indicated concentrations in the absence or presence of 100 μM EG3287. Cell numbers were determined after 3-day incubation.
