# Supplementary material for: Neuropilin-1 antagonism in human carcinoma cells inhibits migration and enhances chemosensitivity
Source: Br J Cancer. 2010 Jan 19;102(3):541–52. doi: 10.1038/sj.bjc.6605539 (PMC2822953; doi:10.1038/sj.bjc.6605539)
Supplement: Supplementary Table 1 [file 6605539x3.doc]

**Supplementary** Table 1. Primer sequences used for real-time quantitative PCR

| Gene | Accession  number | Primer sequence | PCR  product (bp) |
| --- | --- | --- | --- |
| NRP1 | NM_003873 | Forward: 5’-TCC TTG GCA CTG GCT AGT CT-3’  Reverse: 5’-CAA ATC TTC AGA GCC CTT GC-3’ | 203 |
| NRP2 | NM_003872 | Forward: 5’- ATA CCA CAC CAA GGC TGG AG -3’  Reverse: 5’- ACC ACC TAG TCC GGG AGA GT-3’ | 234 |
| GAPDH | NM_002046 | Forward: 5’-GGG GAA GGT GAA GGT CGG AG-3’  Reverse: 5’- CCT GGA AGA TGG TGA TGG GA-3’ | 233 |
